# Impact of ceftolozane/tazobactam concentrations in continuous infusion against extensively drug-resistant *Pseudomonas aeruginosa* isolates in a hollow-fiber infection model

**DOI:** 10.1038/s41598-021-01784-4

**Published:** 2021-11-12

**Authors:** María M. Montero, Sandra Domene-Ochoa, Carla López-Causapé, Sonia Luque, Luisa Sorlí, Núria Campillo, Eduardo Padilla, Núria Prim, Lorena Ferrer-Alapont, Ariadna Angulo-Brunet, Santiago Grau, Antonio Oliver, Juan P. Horcajada

**Affiliations:** 1 Infectious Diseases Service, Hospital del Mar, Infectious Pathology and Antimicrobials Research Group (IPAR), Institut Hospital del Mar d’Investigacions Mèdiques (IMIM), Universitat Autònoma de Barcelona (UAB), CEXS-Universitat Pompeu Fabra, Passeig Marítim 25-29, 08003 Barcelona, Spain; 2 Servicio de Microbiología y Unidad de Investigación, Hospital Son Espases, IdISBa, Palma de Mallorca, Spain; 3 Pharmacy Service, Hospital del Mar, Barcelona, Spain; 4 Laboratori de Referència de Catalunya, Barcelona, Spain; 5 Psychology and Education Science Studies, Universitat Oberta de Catalunya, Barcelona, Spain

**Keywords:** Antimicrobials, Bacteria

## Abstract

Ceftolozane/tazobactam (C/T) has emerged as a potential agent for the treatment of extensively drug-resistant (XDR) *Pseudomonas aeruginosa* infections. As it is a time-dependent antimicrobial, prolonged infusion may help achieve pharmacokinetic/pharmacodynamic (PK/PD) targets. To compare alternative steady-state concentrations (Css) of C/T in continuous infusion (CI) against three XDR *P. aeruginosa* ST175 isolates with C/T minimum inhibitory concentration (MIC) values of 2 to 16 mg/L in a hollow-fiber infection model (HFIM). Duplicate 10-day HFIM assays were performed to evaluate Css of C/T in CI: one compared 20 and 45 mg/L against the C/T-susceptible isolate while the other compared 45 and 80 mg/L against the two C/T-non-susceptible isolates. C/T resistance emerged when C/T-susceptible isolate was treated with C/T in CI at a Css of 20 mg/L; which showed a deletion in the gene encoding AmpC β-lactamase. The higher dosing regimen (80 mg/L) showed a slight advantage in effectiveness. The higher dosing regimen has the greatest bactericidal effect, regardless of C/T MIC. Exposure to the suboptimal Css of 20 mg/L led to the emergence of C/T resistance in the susceptible isolate. Antimicrobial regimens should be optimized through C/T levels monitoring and dose adjustments to improve clinical management.

## Introduction

The indiscriminate use of antibiotics has contributed to the emergence and selection of multidrug-resistant (MDR) and extensively drug-resistant (XDR) bacteria^1,2^ and led to a critical decrease in the availability of alternative antibiotic treatments, limiting treatment options and increasing morbidity and mortality^3^. *Pseudomonas aeruginosa* has an outstanding capacity to develop resistance through a broad range of mechanisms^4–6^. MDR/XDR *P. aeruginosa* isolates are particularly concerning, as they are the leading cause of nosocomial infections and a strong contributor to in-hospital mortality^7^. The ST175 clone is especially significant in several European countries^8^. The development of tailored antimicrobial treatments could greatly improve the clinical management of infections caused by MDR/XDR *P. aeruginosa*.

Ceftolozane/tazobactam (C/T; Zerbaxa; Merck & Co, Inc., Kenilworth, NJ) has emerged as a potential agent against MDR/XDR strains that are resistant to all first-line antibiotics^9^. The combination of ceftolozane, a cephalosporin, and tazobactam, a beta-lactamase inhibitor^6^, has shown promising results in the treatment of infections caused by *P. aeruginosa* strains with different resistance patterns^10^. The current recommended dosage for C/T is a 1-h infusion of 1/0.5 g every 8 h for urinary tract and soft tissue infections and 2/1 g every 8 h for respiratory infections^11^. The pharmacokinetic (PK) properties of ceftolozane have been studied alone and combined with tazobactam in healthy individuals^6,12^. As C/T is a time-dependent antibiotic, the percentage of time during the dosing interval in which free drug plasma concentrations remain above the minimum inhibitory concentration (MIC) (%T_>MIC_) is the best pharmacodynamic (PD) parameter for predicting bacteriological efficacy. The %T_>MIC_ is approximately 40–50% for some cephalosporin^6,12^, but recent studies have shown that the percentage for ceftolozane is much lower, similar to that reported for carbapenems^13,14^. The currently recommended C/T dosing regimen thus might be insufficient against *P. aeruginosa* strains with a C/T MIC above the susceptibility breakpoint of 4 mg/L. Infections caused by these strains would therefore need to be treated with combinations of antibiotics or optimized dosing^15^^.^

The aim of this study was to evaluate different steady-state concentrations (Css) of C/T in continuous infusion (CI) to test the effectiveness of C/T and the emergence of resistance in an in vitro hollow-fiber infection model (HFIM). Three XDR *P. aeruginosa* ST175 isolates with different C/T MICs (2, 8 and 16 mg/L) were tested.

## Material and methods

### Bacterial isolates

Three XDR *P. aeruginosa* clinical isolates were analysed: ST175 (10-023), with a C/T MIC of 2 mg/L; ST175 (09-012), with a C/T MIC of 8 mg/L; and ST175 (07-016), with a C/T MIC of 16 mg/L. These isolates had been previously characterized at a molecular level using pulsed-field gel electrophoresis, multi-locus sequence typing, and whole genome sequencing and are representative of the clones and resistance mechanisms in our environment^16^.

### Antibiotics

C/T (Zerbaxa®; lot number SO15404; expiration date, August 2020) was provided by Merck & Co., Inc. (Kenilworth, NJ). CI C/T dosing regimens were simulated to achieve approximate Css of 20, 45 and 80 mg/L (which respectively correspond to 3, 6 and > 9 g/4.5 g every 24 h)^17^. The exposures to simulate the steady-state human pharmacokinetics of unbound drug were based on elimination half-life of 3 h for ceftolozane^18,19^. A protein-binding estimate was 20% for ceftolozane. The C/T regimens included a dose range based on previously determined C_max_ and AUC^19^. Exposure to tazobactam was not considered, as this drug has a limited role in ceftolozane’s activity against *P. aeruginosa*^20^. C/T concentrations were validated by high-performance liquid chromatography (HPLC)^21^.

### HFIM

The HFIM has been used extensively and described elsewhere^18,22^. Duplicate 10-day HFIM assays were conducted in two stages to investigate the effectiveness of C/T and the development of antimicrobial resistance. Effectiveness was investigated by treating *P. aeruginosa* isolates ST175 (09-012) and ST175 (07-016) with C/T in CI at steady-state concentrations of 45 and 80 mg/L, while resistance was investigated by treating ST175 (10-023) to C/T in CI at steady-state concentrations of 20 and 45 mg/L.

**Figure 1:**
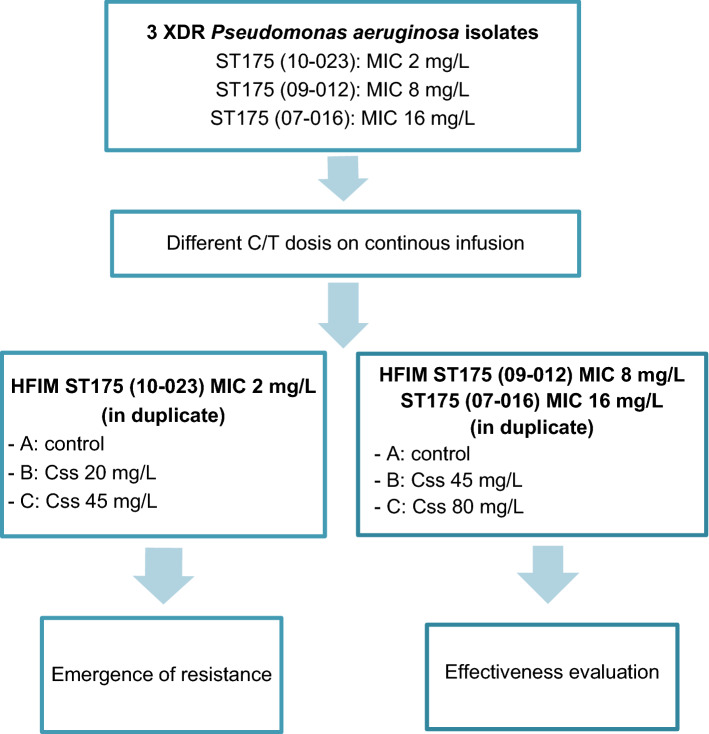
Study flow showing in vitro 10-day HFIM assays conducted with three XDR *Pseudomonas aeruginosa* ST175 isolates with C/T MICs ranging from 2 to 16 mg/L using different Css of C/T in CI: 20 and 45 mg/L to test the emergence of C/T resistance in the susceptible ST175 (10-023) isolate, and 45 and 80 mg/L to test the effectiveness of C/T against the non-susceptible isolates ST175 (09-012) and ST175 (07-016). C/T, ceftolozane/tazobactam; HFIM, hollow-fiber infection model; MIC, minimum inhibitory concentration; XDR, extensively-drug resistant; Css, steady-state concentration; CI, continuous infusion.

Polyethersulfone hemofilters where used as the hollow-fiber cartridges (Aquamax HF03, Nikkiso, Belgium). Each C/T regimen was pumped into the corresponding reservoir by a separate infusion pump to simulate human free drug PK profiles in humans. Fresh drug-free growth medium (cation-adjusted Mueller–Hinton broth [CAMHB]) was continuously infused into the central reservoir to dilute and simulate drug elimination in humans. An equal volume of drug-containing medium was concurrently removed from the central reservoir to maintain an isovolumetric system. The extracapillary space of each HFIM was inoculated with 50 mL of bacterial suspension. High-inoculum infections were simulated. Once inoculated, the bacteria were left in the extracapillary compartment of the HFIM cartridge, where they were exposed to fluctuating drug concentrations. The assays were conducted at 37 °C. Maintenance doses were given continuously at the same rate, according to the clinical dosing frequency. Bacterial densities (log_10_ CFU/mL) in the cartridges were measured at 0, 8, 24, 48, 72, 96, 144, 168, 192 and 240 h. The samples were washed and suspended in saline solution to minimize drug carryover. Serial decimal dilutions were cultured onto drug-free trypticase soy agar (BBL TSA II, Becton Dickinson) plates to determine the total bacterial population. The lower limit of detection (LLOD) was 1.3 log_10_ CFU/mL. Study flow is shown in Fig. 1. Bactericidal activity was defined as a reduction of 3 log_10_ CFU/mL from the initial bacterial load^23^.

### Resistance studies

An aliquot of bacterial suspension from each HFIM was cultured onto drug-containing plates (TSA agar) supplemented with C/T at twofold, fourfold and eightfold the baseline MIC to assess the effect of each regimen on the least susceptible bacterial population. Mutants that grew on these plates were compared with total bacterial population on drug-free TSA plates. When growth was observed after 72 h, up to three colonies were selected to assess C/T MICs and were analysed for changes in MICs from baseline. Antibiotic susceptibility testing was performed according to Clinical & Laboratory Standards Institute (CLSI) guidelines for broth microdilution using CAMHB^24^. The isolates were serially passaged three times on drug-free plates to assess the stability of the phenotype. To investigate the mechanisms leading to C/T resistance, the presence of structural mutations in the catalytic centre of AmpC was analyzed by PCR and sequencing as whole genome sequencing as previously described^25^.

### Drug concentrations

Antibiotic samples were collected at different time points over the first 48 h (0, 3, 5, 7, 9, 23, 25 27, 29 and 47 h) and once a day for the first dose, until the end of the study. Samples were stored at − 80 °C until analysis. All exposures to simulate steady-state human PK of unbound drug were based on the half-life of ceftolozane (exposure to tazobactam was not considered as previously mentioned). Antibiotic concentrations were analysed by HPLC^21^.

## Results

### In vitro* susceptibility and resistance mechanisms*

The isolates had been previously characterized at a molecular level^16^. The ST175 (10-023) isolate was susceptible to C/T (MIC 2 mg/L) and resistant to the other β-lactams due to OprD inactivation and AmpC hyperproduction^16^. The ST175 (09-012) isolate had intermediate resistance to C/T (MIC 8 mg/L) and the mechanisms identified were OprD inactivation, AmpC hyperproduction, and a mutation in PBP3 (R504C) that has been previously associated with increased β-lactam resistance^16^. The ST175 (07-016) isolate was resistant to C/T (MIC 16 mg/L) and in this case the mechanism identified was the production of a class A carbapenemase GES-5 coupled with OprD inactivation^16^.

### HFIM and data analysis

**Figure 2:**
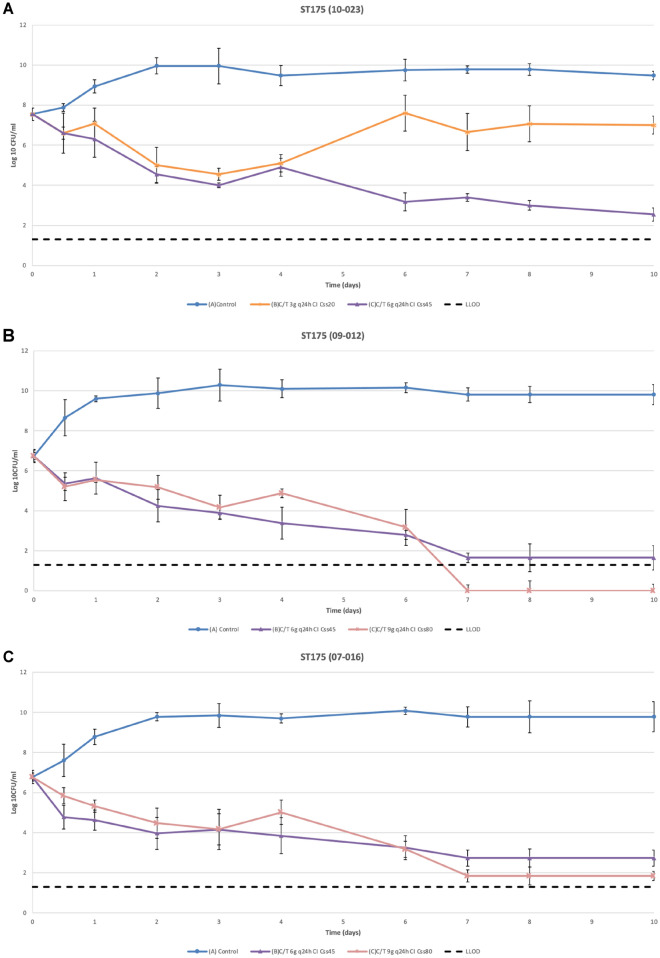
Mean reduction in bacterial density during the 10-day HFIM assays with ST175 (10-023), ST175 (09-012) and ST175 (07-016) isolates treated with different Css of C/T (20, 45 and 80 mg/L) in CI. Respective C/T MIC values of 2, 8 and 16 mg/L. C/T, ceftolozane/tazobactam; CI, continuous infusion; Css, steady-state concentration; LLOD, lower limit of detection; MIC, minimum inhibitory concentration.

Table 1 shows the total mean reduction (log difference at 24 h) for each antibiotic compared with the control. In Fig. 2 results for the reductions in density over time are shown. For ST175 (10-023) the mean bacterial density of the starting inoculum was 7.54 log_10_ CFU/mL (Fig. 2A). A five log_10_ CFU/mL reduction was observed for the Css of 45 mg/L. The Css of 20 mg/L was associated with an initial reduction followed by regrowth on day 6. The final bacterial density was 7 ± 0.45 log_10_ CFU/mL, which corresponds to an overall reduction of 0.54 log_10_ CFU/mL (no bactericidal effect).

**Table 1:** Mean overall reduction in bacterial density (log_10_ CFU/mL ± standard deviation) using alternative C/T Css regimens for each isolate.

	ST175 (10-023)	ST175 (09-012)	ST175 (07-016)
	Log diff day 10^a^	Log diff day 10	Log diff day 10
C/T 3 g q24 h CI Css 20 vs Control	− 2.48 ± 0.14	–	–
C/T 6 g q24 h CI Css 45 vs Control	− 6.94 ± 0.05	− 8.16 ± 0.17	− 7.04 ± 0.06
C/T 9 g q24 h CI Css 80 vs Control	–	− 9.81 ± 0.08	− 7.93 ± 0.10

*C/T* ceftolozane/tazobactam, *CI* continuous infusion, *Css* steady-state concentration.

^a^Log difference at the end of the assay for each regimen compared with the control.

The 10-day HFIM studies to evaluate the effectiveness of higher than the standard Css of C/T (80 mg/L in CI) were performed using the two non-susceptible isolates, ST175 (09-012) and (07-016). The mean starting inoculum was 6.76 log_10_ CFU/mL. Figure 2B, C shows the changes in bacterial density at the different time points analyzed. Overall, the C/T Css of 80 mg/L in CI showed a slight advantage over the Css of 45 mg/L. Both dosing regimens showed similar effectiveness against ST175 (09-012) up to day 6, but on day 7, the higher regimen achieved eradication of the bacterial population (below the LLOD). The curve for the Css of 45 mg/L plateaued and the final density was 1.65 ± 0.6 log_10_ CFU/mL. A similar pattern was observed for ST175 (07-016), but in this case, the Css of 80 mg/L did not eradicate the bacterial population. In brief, C/T at both Css (45 and 80 mg/L) exerted bactericidal activity against the two non-susceptible isolates.

### Resistance studies

**Figure 3:**
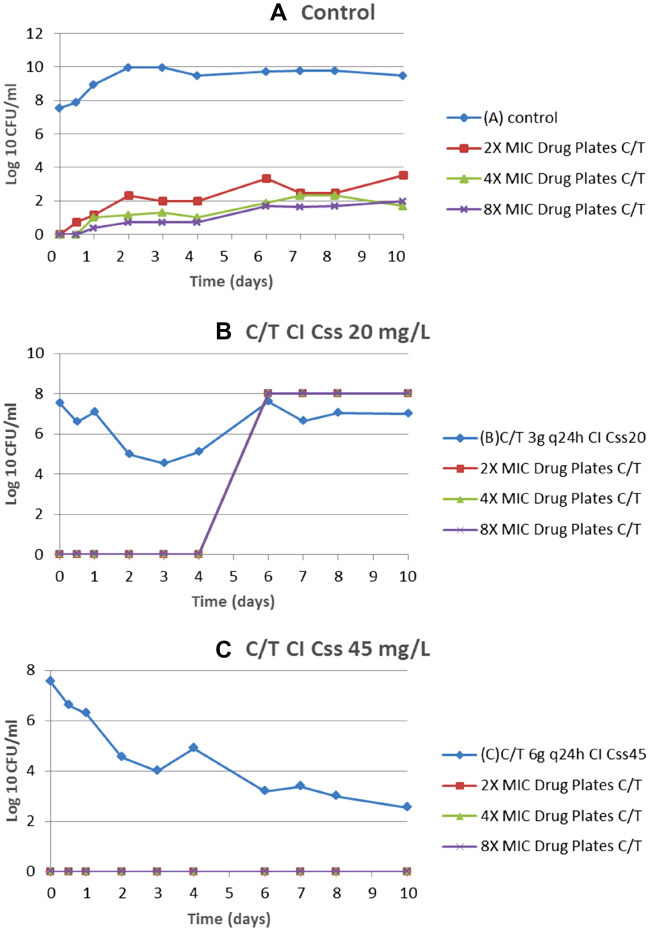
Emergence of C/T resistance in the ST175 (10-023) isolate using Css of 20 and 45 mg/L in CI. C/T, ceftolozane/tazobactam; CI, continuous infusion; Css, steady-state concentration; MIC, minimum inhibitory concentration.

In the 10-day HFIM, a C/T-resistant subpopulation emerged in the susceptible isolate after exposure to the C/T Css of 20 mg/L. Resistance emerged on day 6 at concentrations of 2-, 4-, and eightfold the MIC, and resulted in 1 CFU/mL in 1.6 × 10^9^, 7.8 × 10^10^, and 3.9 × 10^10^, respectively. The C/T MIC was ≥ 256 mg/L. Compared to the original population, the resistant subpopulation had a lower meropenem MIC (8 vs 16 mg/L) and a lower imipenem MIC (2 vs 8 mg/L). The analysis of mutations within a set of genes involved in antibiotic resistance compared with those already present in the parental isolate (sequencing of *bla*_AmpC_ gene) revealed a 19-amino acid deletion (K232-G250) in the Ω-loop of AmpC, which has been associated with C/T resistance^25^*.* No resistant subpopulations were detected following exposure to C/T in CI at a Css of 45 mg/L (Fig. 3).

### Drug concentrations

The relationship between observed and predicted C/T concentrations is shown in the Supplemental Material. We assessed the agreement between observed results and predicted results with the Bland–Altman plot. For Css 45 and 80 mg/L difference values have less than a 1.96 of standard deviation (SD) from the mean. On the other hand, for Css 20 mg/L one of the 15 values deviates slightly from 1.96 SD.

## Discussion

Optimization of antibiotic treatments based on PK/PD properties is essential in MDR/XDR *P. aeruginosa* infections. C/T has emerged as a promising option in this setting^18^. The standard C/T dose in intermittent infusion regimens can be optimized in high-inoculum infections, but CI may be a better option for achieving PK/PD targets. Our HFIM study compared different Css of C/T in CI against three XDR *P. aeruginosa* ST175 isolates with C/T MICs ranging from 2 to 16 mg/L. The criteria for selecting C/T dosages were to compare lower and higher Css from different doses of C/T that are recommended for difficult-to-treat infections. The ST175 clone was selected because it is the most prevalent in our environment and it has been associated with MDR/XDR isolates involved in nosocomial infections^8^.

Few studies have evaluated C/T infusion dosing in the clinical practice. Pilmis et al.^26^ compared intermittent infusion and CI in patients with MDR *P. aeruginosa* infections, and concluded that the current recommended dosing regimen provided unsatisfactory coverage. Another Monte Carlo simulations found that extending the duration of C/T infusion improved the probability of target attainment in the treatment of infections caused by MDR *P. aeruginosa* strains with different C/T MICs in patients with different renal functions levels^15^. These results are in consonance with our previous experiments in which different type of infusion (1 h, 4 h and CI) were examined against the same three *P. aeruginosa* isolates. In summary, these studies showed that the CI regimen achieved the highest bacterial reduction even against non-susceptible isolates (an overall reduction of − 4.95 log_10_ CFU/ml for the CI infusion versus a reduction of − 1.87 and − 2.78 for the 1 h and 4 h infusion, respectively)^27^. Sime et al.^11^ described the population PKs of unbound C/T and evaluated the adequacy of recommended dosing regimens in critically ill patients without renal impairment. They concluded that a loading dose of 1 g/0.5 g followed by 3 g/1.5 g in CI was adequate for empirical coverage of a T > MIC target of 100%. In our study, however, CI of 3 g of ceftolozane resulted in the emergence of C/T resistance, indicating that dosing according to PK/PD parameters is important for improving clinical management.

The administration of C/T at a Css of 45 mg/L achieved a reduction in bacterial density in both susceptible and resistant isolates. It also exerted a bactericidal effect regardless of the C/T MICs of the isolates. This sustained suppression of bacterial growth suggests that C/T in CI may achieve concentrations above the susceptibility breakpoint for a longer period of time. This would be particularly important for *P.* aeruginosa isolates with higher C/T MIC values.

Optimization of Css in a CI regimen is necessary to achieve an optimal therapeutic effect. A retrospective study analyzing the performance of C/T in patients with XDR *P. aeruginosa* infections, most of whom were receiving CI, found that 66% achieved supratherapeutic levels^28^. These results highlight the importance of monitoring C/T plasma concentrations when aiming to optimize treatment^28^. We performed a 10-day HFIM study to determine whether a low CI C/T dosing regimen would be as effective as the standard regimen against the C/T-susceptible isolate ST175 (10-023) or possibly contribute to the selection of C/T-resistant subpopulations. We found that a Css of 20 mg/L clearly failed to prevent the emergence of resistance, whereas a Css of 45 mg/L had a bactericidal effect. Sequencing of *bla*_AmpC_ gene in the resistant subpopulation that emerged revealed a 19-amino acid deletion (K232-G250) in the Ω-loop of AmpC, supporting previous reports of a link to C/T resistance^25^. The C/T-resistant subpopulation was also associated with a decrease in meropenem and imipenem MIC values. This phenomenon of partial reversal of carbapenem resistance concomitant with the acquisition of C/T resistance has been previously reported^25^. Bacterial antibiotic susceptibility is therefore dynamic and may be influenced by a gain of resistance in other antibiotics^29^.

Infections caused by *P. aeruginosa* isolates with C/T MIC values above 2 mg/L have been associated with poor outcomes when treated with a standard C/T dosing regimen^30^. The use of higher doses (up to 6 g/3 g every 24 h), mainly against non-susceptible strains, has not been found to produce adverse effects^11^. In this context, we evaluated a CI C/T regimen with a Css of 80 mg/L as an option for optimizing the treatment of infections caused by resistant *P. aeruginosa* strains. Our results showed that this higher dose displays a slight advantage than the currently recommended regimen, particularly in the case of the isolate with the intermediate C/T MIC, in which the eradication of the bacterial population was achieved.

This study had some limitations. First, we only studied three *P. aeruginosa* isolates, although they are representative of the different ranges of C/T susceptibility in our environment. Second, despite the use of clinical parameters, we were unable to examine toxicity and infection site effects in vitro, or to determine the contribution of the immune system to bacterial killing, as host immunity could, to a certain extent, modify PD targets. Nonetheless, this absence of immunity means our findings can be extrapolated to immunocompromised patients. Finally, it should be clarified that exposure to tazobactam was not considered although it has a limited role in ceftolozane’s activity against *P. aeruginosa.*

In summary, our results show that C/T in CI at Css of 45 mg/L leads to a decrease in bacterial burden in *P. aeruginosa* and is useful against non-susceptible isolates (with a MIC of 8 and 16 mg/L). CI at a Css of 80 mg/L had the strongest bactericidal effect. Administration of the suboptimal Css of 20 mg/L resulted in the emergence of C/T resistance in the susceptible isolate (MIC 2 mg/L). Antimicrobial regimens can be individually optimized by adjusting antibiotic doses to both C/T MIC values and PK/PD targets. Therapeutic drug monitoring would favour better clinical management and help prevent the emergence of antibiotic resistance.

## Supplementary Information

Supplementary Information.
