# Supplementary material for: Impact of ceftolozane/tazobactam concentrations in continuous infusion against extensively drug-resistant Pseudomonas aeruginosa isolates in a hollow-fiber infection model
Source: Sci Rep. 2021 Nov 12;11:22178. doi: 10.1038/s41598-021-01784-4 (PMC8589991; doi:10.1038/s41598-021-01784-4)
Supplement: Supplementary file 1 — Supplementary Information. [file 41598_2021_1784_MOESM1_ESM.pdf]

Impact of ceftolozane/tazobactam concentrations in continuous infusion against extensively drug-resistant *Pseudomonas aeruginosa* isolates in a hollow-fiber infection model

Scientific Reports

María M Montero[1], Sandra Domene-Ochoa[1], Carla López-Causapé[2], Sonia Luque[3], Luisa Sorlí[1], Núria Campillo[3], Eduardo Padilla[4], Núria Prim[4], Lorena Ferrer-Alapont[1], Ariadna Angulo-Brunet[5], Santiago Grau[3], Antonio Oliver[2], Juan P Horcajada[1]

1. Infectious Diseases Service, Hospital del Mar, Infectious Pathology and Antimicrobials Research Group (IPAR), Institut Hospital del Mar d'Investigacions Mèdiques (IMIM), Universitat Autònoma de Barcelona (UAB), CEXS-Universitat Pompeu Fabra Barcelona, Spain
2. Servicio de Microbiología y Unidad de Investigación, Hospital Son Espases, IdISBa, Palma de Mallorca, Spain
3. Pharmacy Service, Hospital del Mar, Barcelona, Spain
4. Laboratori de Referència de Catalunya, Barcelona, Spain
5. Psychology and Education Science Studies, Universitat Oberta de Catalunya, Barcelona, Spain.

Corresponding author: María Milagro Montero

Supplementary Material.

Table S1. Observed versus predicted antibiotic concentrations achieved during each HFIM study.

	**Css (mg/L) ± SD**	
**Dosing regimen**	**Predicted value**	**Observed value**
**C/T 3 g q24 h CI**	20	20.91 ± 1.68
**C/T 6 g q24 h CI**	45	47.29 ± 5.43
**C/T 9 g q24 h CI**	80	82.98 ± 8.18

Data are presented as the mean concentration ± standard deviation. Css, steady-state concentration; CI, continuous infusion.

Figure S1. Bland-Altman plot between observed and targeted ceftolozane concentrations for the three regimens in the overall experiments: Css of 45 mg/L, Css of 20 mg/L and Css of 80 mg/L.

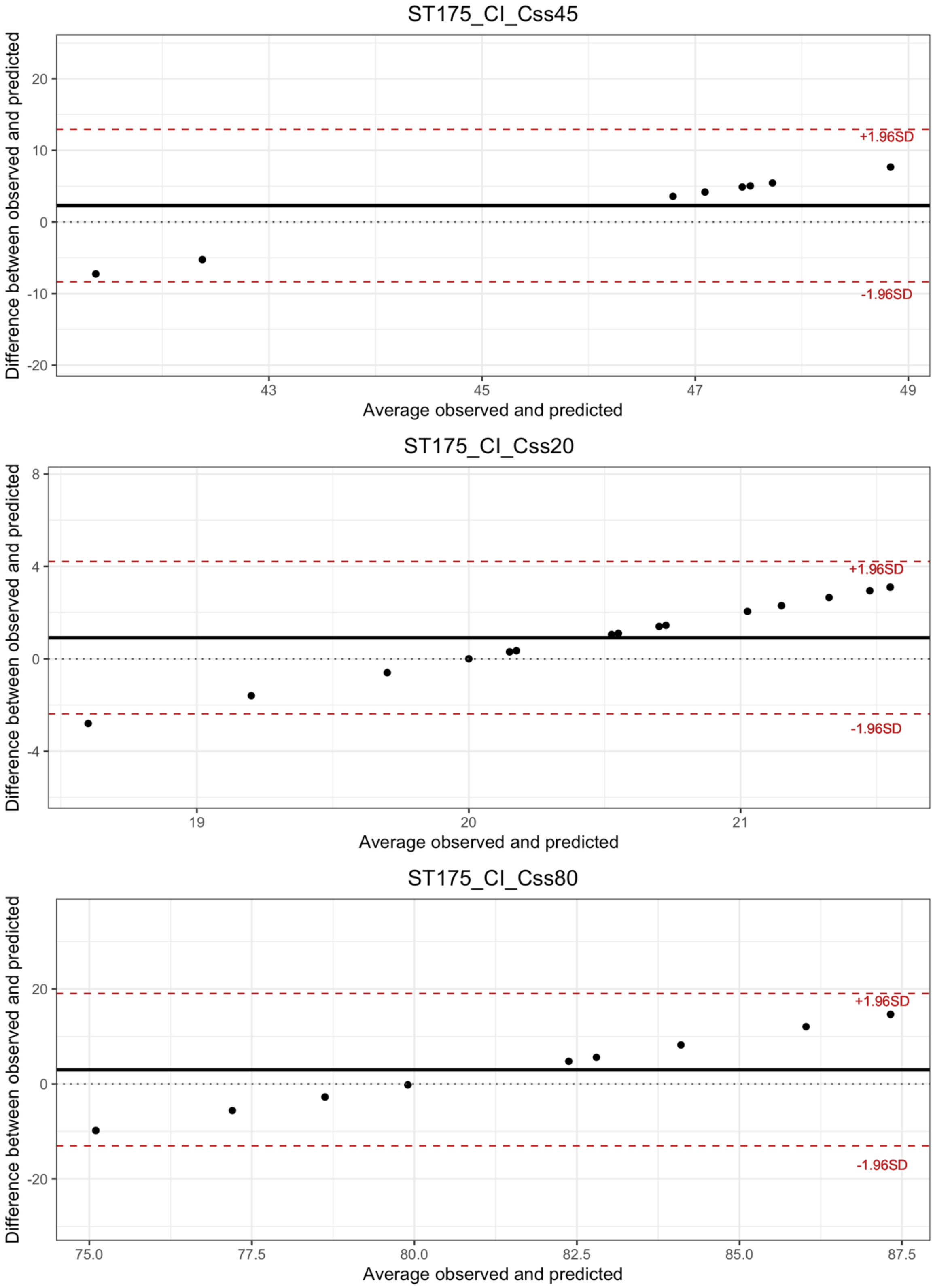

Abbreviations: inf, infusion; CI, continuous infusion; Css, steady-state concentration.
